# Task-evoked pupil responses during free-viewing of hierarchical figures in relation to autistic traits in adults

**DOI:** 10.1038/s41598-025-92904-x

**Published:** 2025-03-21

**Authors:** Chloe Brittenham, Antoinette Sabatino DiCriscio, Vanessa Troiani, Yirui Hu, Jennifer B. Wagner

**Affiliations:** 1 Departments of Pediatrics and Neuroscience, Albert Einstein College of Medicine and Montefiore Medical Center, Bronx, NY USA; 2 Department of Psychology, The Graduate Center, City University of New York, New York, NY USA; 3 Department of Psychology, College of Staten Island, City University of New York, Staten Island, NY USA; 4 Geisinger Autism and Developmental Medicine Institute, Geisinger Health System, Lewisburg, PA USA; 5 Department of Biomedical and Translational Informatics, Geisinger, Geisinger Health System, Danville, PA USA

**Keywords:** Pupillometry, Pupil, Hierarchical visual processing, Global processing, Local processing, Autistic traits, Human behaviour, Disability

## Abstract

Sensory processing differences, particularly within the visual domain, are common in neurodevelopmental conditions, including autism. Studies examining hierarchical processing of figures containing global (i.e., gist) and local (i.e., detail) elements are inconsistent but converge on a common theme in relation to autism: slowed global processing and a locally-oriented default. We examined behavioral and pupillary responses in adults with varying levels of autistic traits during a free-viewing hierarchical processing task. Results showed that participants were both more likely and faster to report global elements, but contrary to our hypothesis, differences in level of autistic traits were unrelated to spontaneous reporting of global vs. local elements. When examining phase-based analysis of pupillary responses, participants high on autistic traits showed more early and less later constriction within trials. Further, trajectory-based pupillary analysis revealed two trajectories, one characterized by constriction and the other dilation, and results showed that the dilation group disproportionately included low traits individuals. Findings suggest that although high and low traits groups showed similar behavioral responses, visual strategies used may differ, as indicated by pupillometry. This study advances our understanding of the relationship between autistic traits and visual processing, laying groundwork for further investigations into neurodivergent visual processing mechanisms.

## Introduction

The fast and dominant perception of a global element over local detailed elements when viewing hierarchical visual stimuli has been found repeatedly within the general population. This phenomenon is sometimes referred to as global precedence^1^ and/or global default^2^. Deviations in this pattern of hierarchical processing have been found in individuals with autism and other related neurodevelopmental differences^3–5^.

Beginning with Shah and Frith^6^, research spanning four decades has explored these hierarchical processing differences in autism, with some suggesting that they may play a role in core autism features^7–9^. Overall, past work converges to suggest that in autism, global processing may be slowed^5,10^, hierarchical processing may be locally oriented by default^11^, and local processing may be superior, facilitated by this local default^12^, although there are also some inconsistent findings^4,13^.

Autistic traits also vary at subclinical levels within the general population^14^, and there exists a robust positive association between sensory processing differences and number of autistic traits within individuals who do not have an autism diagnosis^15^. Despite these associations, research exploring the link between global/local processing differences and autistic traits in the general population is limited. While some studies have found global and/or local processing differences to be associated with autistic traits^16,17^, studies using hierarchical stimuli have yielded mixed results^18,19^.

**Fig. 1:**
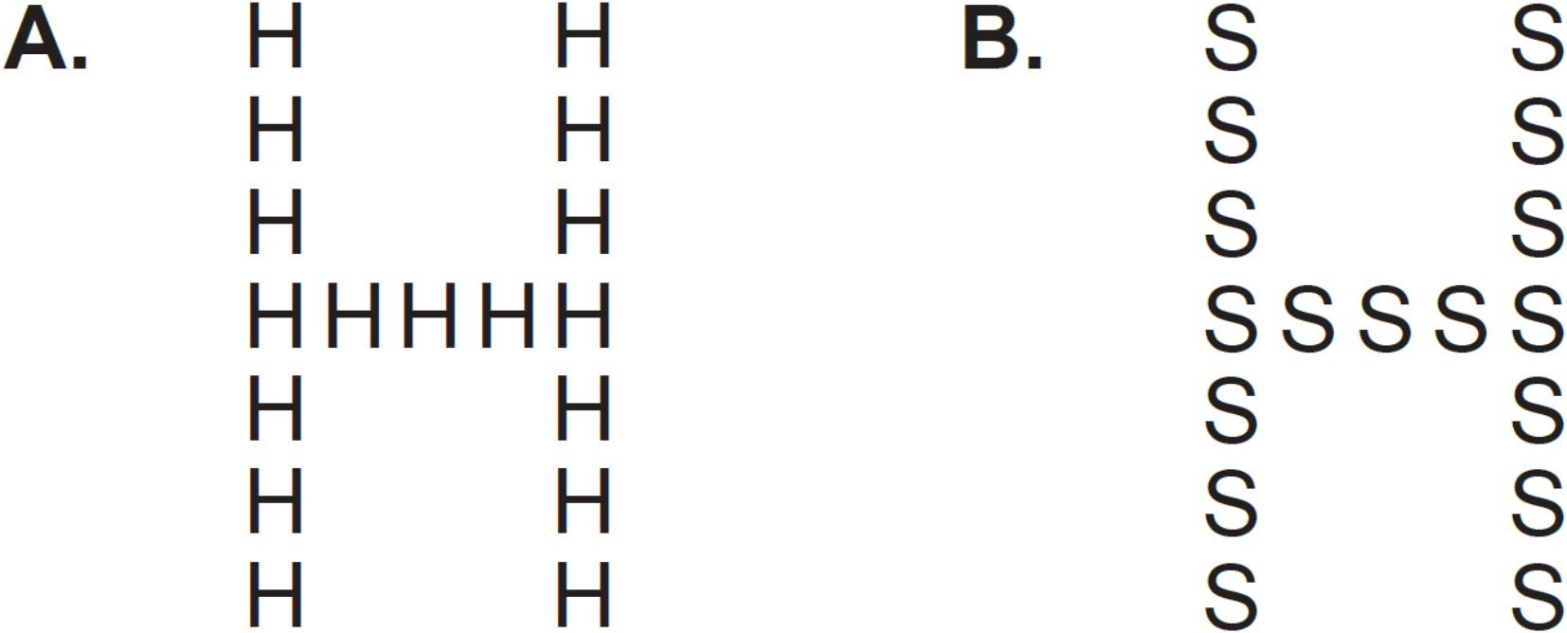
Examples of traditional (**A**) congruent and (**B**) incongruent hierarchical stimuli. These tests often involve the presentation of hierarchical figures in which a larger figure (traditionally a letter) is composed of smaller letters or figures. Stimuli can be either congruent (the larger letter [global level] is the same as the smaller letters [local level]) or incongruent (the larger [global level] and smaller letters [local level] are different from one another. Performance is often measured through reaction time, speed to report global/local level, and/or in accuracy of reporting the global/local level.

These inconsistencies may stem, in part, from confounding factors in traditional hierarchical stimuli tasks, ranging from task type (e.g., selective or divided attention), element size, salience of stimulus elements, and/or spatial frequency differences^2,20–22^. For example, the Navon figure test^1^ (see Fig. 1) has been critiqued for a lack of control over the spatial frequency composition of the stimuli, with global information encoded in low spatial frequencies^2^. Thus, any observed global precedence using the Navon test could be due to low-level visual information processing rather than a conceptual ‘gestalt’ interpretation. A study by Campana et al.^2^ controlled for these confounding factors using stimuli that required processing of local elements before global elements. The development and use of these more tightly controlled stimuli may be important for understanding the underlying basis of visual hierarchical processing differences in those with autism, especially since low-level visual processing differences, such as atypical high and low spatial frequency processing, have also been found to be linked with autism diagnosis and autistic traits^19,23,24^.

Hierarchical visual processing has also been studied using infrared eye-tracking technology, which can allow for a more objective analysis of subtle differences in visual processing. Although most eye-tracking studies in the autism literature focus on social attention^25–28^, a few have explored global/local processing in autism^29,30^. One metric captured by infrared eye trackers is pupil size, and the measurement of pupil size modulation, or pupillometry, is increasingly being used as a metric of interest^31^. The pupillary response is a complex signal resulting from contributions from several underlying factors, including the luminance of what is being attended to, heightened attentional recruitment and/or cognitive load, and attentional scope (see^32^). Past work by DiCriscio and colleagues^30,33,34^ has shown that the Navon figure test might elicit different patterns of pupil responses for global versus local processing. Importantly, this work emphasized that traditional pupillary analysis methods may mask rapid changes in pupil diameter. Therefore, instead of employing a data reductionist approach as is commonly done in pupillometry, DiCriscio et al. treated the pupil response akin to a neural waveform, examining phasic changes^34^ and later examining group trajectory patterns through latent class growth modeling^30,33^.

In analyzing phasic changes in adults during a Navon figure task, DiCriscio et al.^34^ found greater pupil constriction and re-dilation when attending to local versus global information, suggesting a narrowed attentional focus for local details in the early part of the response. Further, this group found evidence that patterns of pupil responses vary with autism diagnosis in children^30^ and with different levels of autistic traits in adults^33^, suggesting differences in visual processing strategies and/or arousal levels associated with visual attention^30,33^. Their findings underscore the nuanced relationship between pupillary dynamics and cognitive/attentional processes and provide an important foundation for the exploration of hierarchical visual processing through examination of the task-evoked pupil response as it relates to autistic traits.

An additional area for consideration from past studies is the use of hierarchical figure paradigms that employ goal-directed tasks where participants are instructed to either identify the global or the local element of the hierarchical figure (e.g.,^30,33,34^). Other studies have found that examining the spontaneous processing of these figures can also reveal meaningful information. Koldewyn et al.^11^ found that autistic children respond with the local element of a stimulus in the initial trials of a free-viewing task, suggesting a local default style of processing. Turi et al.^35^ investigated pupillary responses during dynamic global/local perception tasks and found associations between pupil modulation and attentional strategies, indicating a preference for local processing in individuals with higher levels of autistic traits. Importantly, explicit task instructions (as given in goal-directed conditions) caused these associations to disappear. These findings highlight the importance of free-viewing paradigms in understanding default processing patterns.

The goal of the current study is to elucidate the potential sources of differences in hierarchical processing linked to autistic traits, which may play a role in both superior local visuospatial skills and social communication difficulties, as aberrant hierarchical processing may disrupt the ability to rapidly deduce or extract global elements—or the “gist”—which is essential for synthesizing and generalizing information in social contexts^9,36^. Here, we use the hierarchical stimuli from Campana et al.^2^ and examine both behavioral performance and pupil waveforms in individuals with high levels of autistic traits. We anticipated that participants with low levels of autistic traits would spontaneously report the global orientation faster and more often than the local orientation and those with high levels of autistic traits would have more default reports of the local orientation. For pupillary responses, we hypothesized that the high traits group would show greater constriction in the early part of the pupil response as compared to the low traits group, reflecting a narrower attentional scope (see^34^) and local default. Alternatively, if the high traits group is showing greater recruitment of visual attention in general (see^33^), we might expect greater dilation overall.

## Method

### Justification for sample size

A power analysis based on prior findings^33^ determined that 26 participants per group (*N* = 52) are needed to achieve 85% power for detecting medium-to-large effects (*d* =  − 0.85) with *α* = 0.05 in between-group differences in pupil trajectory response group membership. Additionally, a power analysis informed by a study of default processing in autistic and nonautistic children^11^ indicated that 60 participants would be required to achieve 85% power for detecting significant correlations (*α* = 0.05) between SRS-2 scores and a local preference. The larger sample size in this study ensures robust statistical power for detecting effects across multiple outcomes, enables stratified comparisons based on autistic trait levels in the broader autism phenotype, and supports the analysis of continuous effects of autistic traits. This design enhances generalizability and facilitates not only behavioral analyses as in Koldywn et al.^11^, but also the analysis of pupil response trajectories during spontaneous hierarchical processing—a context not previously explored, as DiCriscio et al.^33^ focused on directed global and local tasks.

### Participants

One hundred and thirty-eight adults with normal or corrected to normal vision (see below) were recruited through the City University of New York (CUNY) and participated in the current study. Informed consent was obtained from all participants, and the protocol adhered to the principles of the Declaration of Helsinki. All procedures were approved by the Institutional Review Board of the City University of New York.

Of the 138 participants, 120 contributed sufficient data (see behavioral data processing section) and were included in the reported analyses. The included sample of adults ranged in age from 18 to 58 years (*M*_age_ = 24.14 years, *SD* = 7.47) and participated either at the College of Staten Island (*n* = 49) or The Graduate Center (*n* = 71). Preliminary analyses considered age as a covariate given the wide age range, but no significant age effects were found for general behavioral and pupillary analyses.

Fifty-seven participants wore vision correction (contacts: *n* = 16; glasses: *n* = 41). Of the final sample of participants who self-reported demographic information, 75% (*n* = 90) identified their ethnicity as not Hispanic or Latinx, and 25% identified as Hispanic or Latinx (*n* = 30). Racially, 45.8% identified as White or Caucasian (*n* = 55), 20% as Black or African American (*n* = 24), 17.5% as Asian (*n* = 21), 15.8% as multiracial or other (*n* = 19), 5.8% as American Indian or Alaskan Native (*n* = 7), and 0.8% as Native Hawaiian or Pacific Islander (*n* = 1). Approximately sixty percent (59.2%) identified as female (*n* = 71), 36.7% as male (*n* = 44), and 4.2% as non-binary (*n* = 5).

A small number of included participants (*n* = 7; 5.8%) self-reported an autism spectrum diagnosis. Because analyses remained unchanged with and without the inclusion of these participants, all reported analyses include this small self-identified autistic sample.

### Materials and apparatus

Visual acuities were measured by the experimenter using a Snellen eye chart at 20 ft. distance (see Table 1). An SR Eyelink 1000 Plus eye tracker (https://www.sr-research.com/) recorded gaze and pupil size at 1000 Hz while participants completed a hierarchical figure task adapted from Campana et al.^2^ Participants were seated 79 cm from a 24-inch ASUS VG248QE LCD screen (refresh rate 144 Hz, resolution: 1920 × 1080 pixels, size of the screen: 569.4 × 340.4 mm) and gaze was stabilized using a chin rest. Ambient light levels at both testing locations were measured using a REED, Precision Instrument (https://www.reedinstruments.com/): 660 Lux; 61.32 Fc at the College of Staten Island and 290 Lux; 26.94 Fc at The Graduate Center. Although lighting conditions at both locations were similar, it was not possible to control each setting to the exact lux level. Reaction time (RT) and accuracy were recorded through keyboard button presses.

**Table 1:** Participant characteristics for the full sample (*n* = 120).

	%	Mean	*SD*	Range
Gender				
Female	59.20			
Male	36.70			
Non-binary	4.20			
Age		24.14	7.47	18–58
Visual Acuity at 20 ft (20/*x*)		20/18.77	4.03	20/13–20/25
SRS-2 total T score		59.20	10.54	40–90
Awr T		54.45	10.70	32–86
Cog T		58.40	9.34	42–90
Com T		58.83	11.10	41–90
Mot T		59.59	10.76	41–90
RRB T		58.89	12.20	40–90
SCI T		58.04	10.10	40–90

Abbreviations: SRS-2, Social Responsiveness Scale, 2nd Edition; Awr T, Social Awareness t-score; Cog T, Social Cognition t-score; Com T, Social Communication t-score; Mot T, Social Motivation t-score; RRB T, Restricted Interests and Repetitive Behaviors.

### Stimuli

**Fig. 2:**
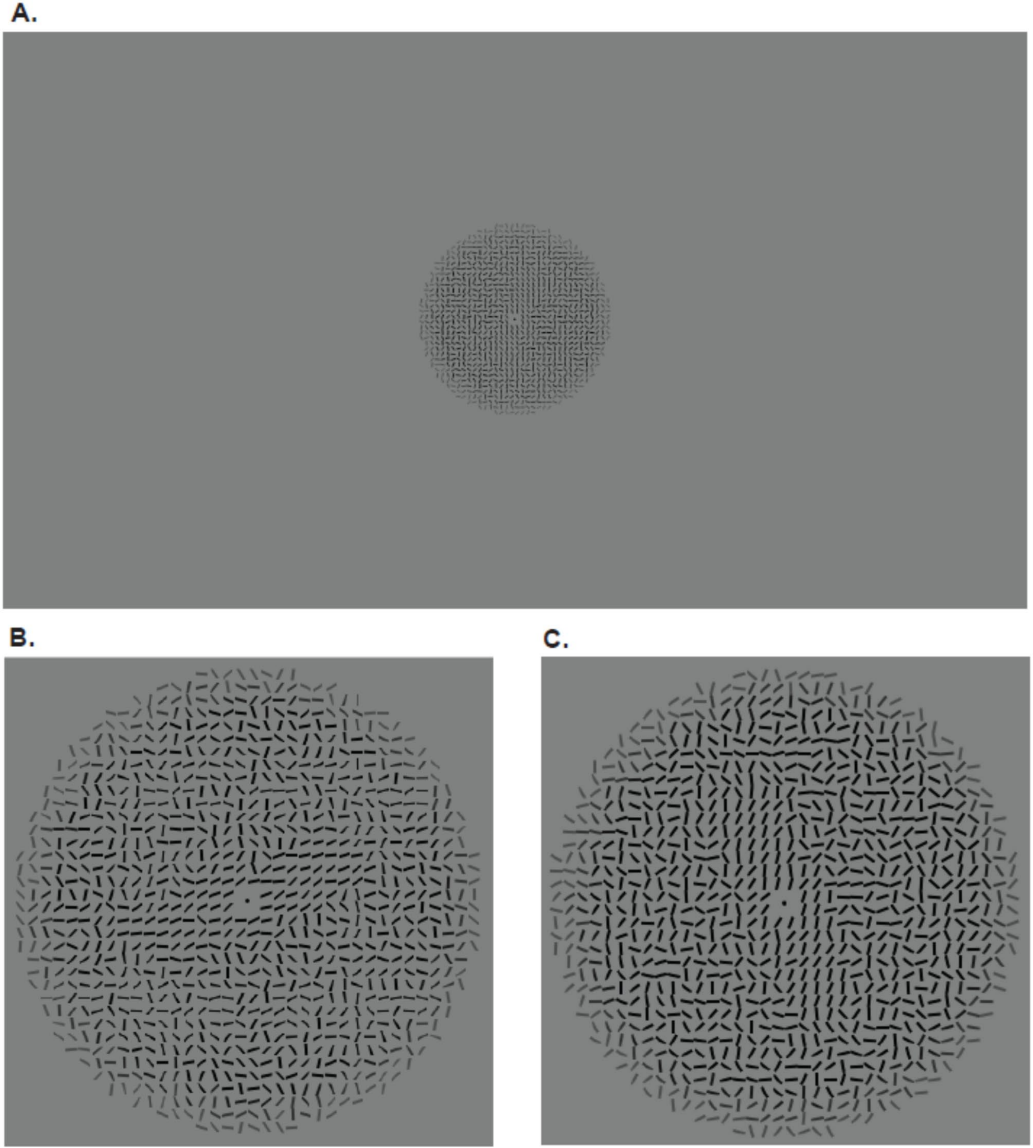
(**A**) Example of an incongruent stimulus as it was displayed on the full screen. Example of a (**B**) congruent stimulus and (**C**) an incongruent stimulus, zoomed in.

Stimuli were circular textures (7.4 degrees of visual angle) composed of oriented black lines on a gray background adapted from Campana et al.^2^, see Fig. 2A. Lines were 0.18 degrees in visual angle, and there was a distance of 0.2 degrees of visual angle between the center of the lines. The radiuses of the circular textures were 36 lines in length. Organization of the lines within the circular textures was fully randomized apart from a rectangular area (4 × 18 lines) in the center surrounding a fixation dot where the lines were approximately aligned to a specific orientation, which was the local orientation. The overall tilt of this region defined the global orientation. Stimuli could be either congruent, meaning the global orientation was the same as the local orientation (see Fig. 2B), or incongruent, meaning the global and local orientations were different (see Fig. 2C).

Based on Campana et al.^2^, both global and local orientations were constrained to four possible orientations: − 67.5, − 22.5, 22.5, and 67.5. Negative values indicate a tilt to the left of a vertical reference line. In incongruent stimuli, global and local orientations within the stimulus were always at an angular distance of 45 degrees or − 45 degrees to ensure that participants would not be able to determine one from the other. This resulted in eight possible combinations of global and local orientations for incongruent stimuli and four types of congruent stimuli. Within the rectangular (global) shape, a line’s orientation was determined through selection from a von Mises distribution:

$$f\left( {x\backslash \mu ,k} \right) = \frac{{e^{k\cos (x - \mu )} }}{2\pi I(k)}$$

where *I* is a modified Bessel function of the order of 0, and the two parameters are *μ* (the mean) and *k* (the inverse of the variance) of a normal distribution. A large coherence value (*k*) results in a steep distribution where all lines that make up the global shape are similar to the mean orientation value (*μ*), and the lines appear to be very closely aligned with one another. When *k* = 0, the lines are randomly oriented. For this study, coherence was fixed across stimuli and reflected the highest coherence level (*k* = 5) used in Campana et al.^2^.

**Fig. 3:**
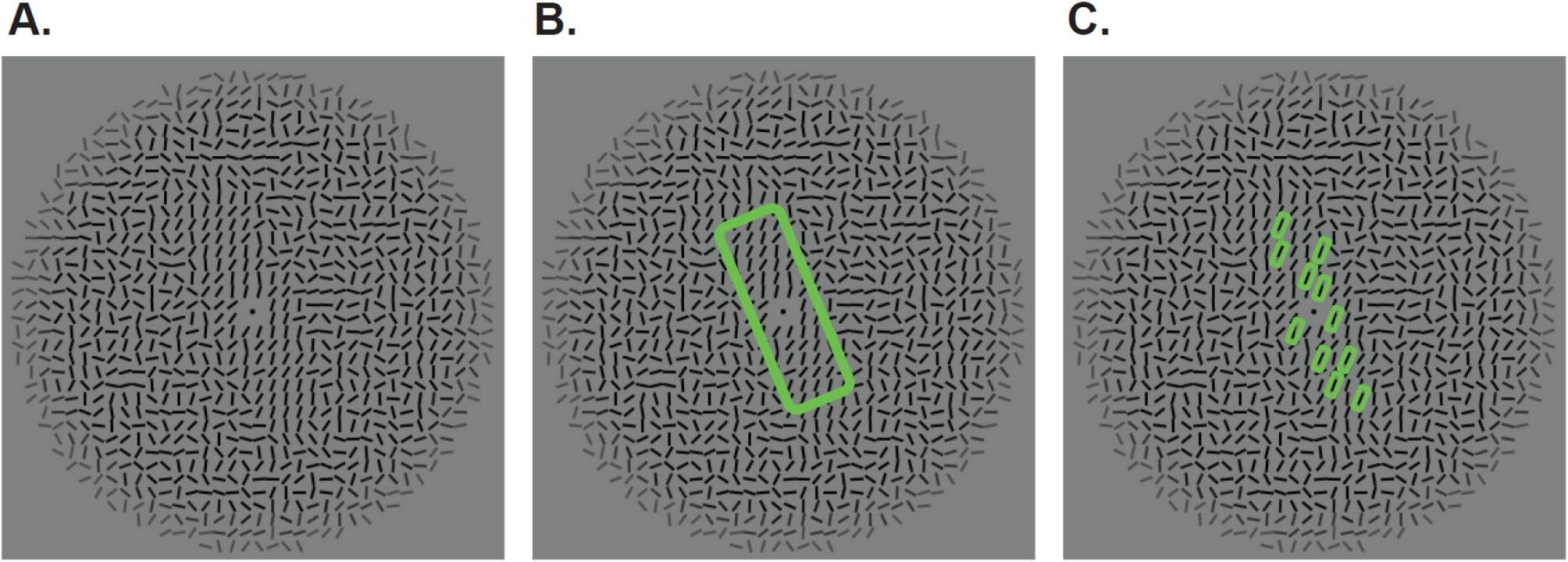
(**A**) Sample stimulus. (**B**) Large green rectangle highlighting the orientation of the global shape. (**C**) Small green rectangles highlighting the overall orientation of the homogenous local lines.

Stimuli were created in MATLAB Version 9.11^37^ using an adaptation of a script written for use in Campana et al.^2^ (see Fig. 3A). The local orientation (*μ*) was chosen from one of the four possible angles. The rectangular area that formed the global orientation was positioned as + /- 45 degrees from *μ*. A “buffer zone” was created, which effectively “smoothed” the border of the rectangular shape. This was done by creating intermediate coherence values where the value of *k* (coherence parameter) gradually decreased from its maximal value (*k* = 5) to zero (completely random orientations) over three lines. Figure 3 shows an example stimulus (Fig. 3A) with the global orientation highlighted (Fig. 3B) and the local orientation highlighted (Fig. 3C).

### Procedure

**Fig. 4:**
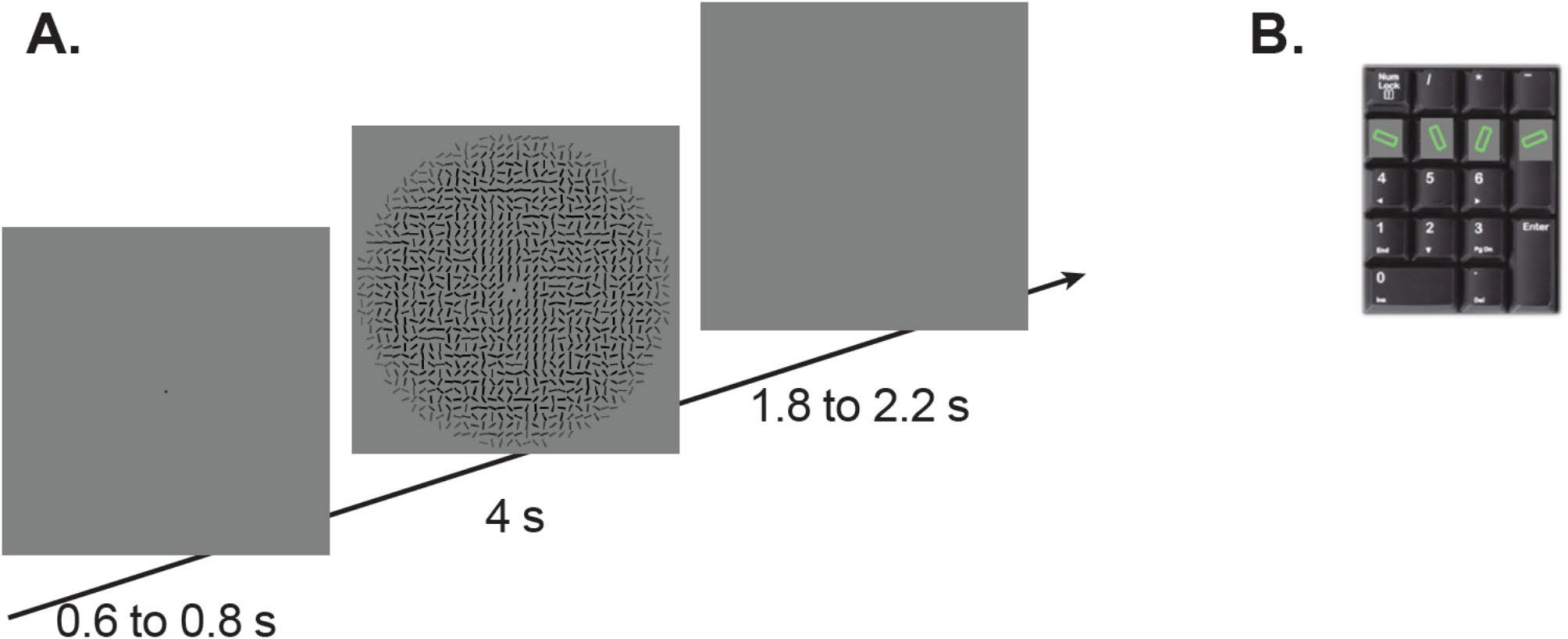
(**A**) Schematic diagram of trials. (**B**) Response options on the participants’ keyboard. Stimuli and tasks adapted from Campana et al. (2016).

After giving informed written consent, participants’ visual acuity was tested monocularly and binocularly (Table 1). During the experiment, a chin rest was used to stabilize the head, maintaining a constant distance between the eyes and the eye-tracker. Nine-point calibration and validation were performed before the experimental tasks. Following initial difficulties during pilot testing with calibrating individuals wearing eyeglasses, we optimized acquisition in participants wearing eyeglasses by ensuring their glasses were cleaned prior to calibration and ensuring that the angle of the infrared tracker was not aligned with the plane of the lenses to avoid glare. As part of a set of eye-tracking tasks, participants completed a free-viewing task with these hierarchical stimuli during which they reported their default perception of the orientation. All trials began with a fixation point presented for 0.6–0.8 s, then the stimulus was presented for 4 s, followed by an intertrial interval of 1.8–2.2 s (see Fig. 4A), and participants responded by key press (see Fig. 4B).

Before the experimental block, participants performed 12 trials of practice, during which they responded with their default perception of congruent stimuli (orientations of local and global level were identical). Participants were told that there was no correct answer and to report their impression. Participants were asked to select one of the four orientations by pressing a key (see Fig. 4B) and to respond as soon as they knew their response, while the stimulus was still on the screen. If they did not see an orientation, they were told to press either the up or down arrow. Following practice, they responded to both congruent stimuli and incongruent stimuli, where the orientations of local and global level were the same or different, respectively (48 total trials; 24 congruent, 24 incongruent).

Following the eye-tracking tasks, participants completed questionnaires assessing demographics, autism diagnosis, family history, and autistic traits. The free-viewing task lasted approximately 15–20 min, and the questionnaires took about 20 min to complete.

### Assessments of autistic traits

Autistic traits were measured using the Social Responsiveness Scale, Second Edition (SRS-2^38^) self-report Adult Form (see Tables 1 and 2). This standardized questionnaire comprises 65 questions, quantifying traits and severity of autism spectrum disorder (ASD) symptoms; social communication difficulties and restricted and repetitive behaviors. It consists of five subscales targeting distinct aspects of social responsiveness and behaviors: Social Awareness (Awr), Social Cognition (Cog), Social Communication (Com), Social Motivation (Mot), and Restricted Interests and Repetitive Behavior subscales (RRB). Total T-scores on this scale which exceed 59 indicate mild to severe concerns for autism^38^.

**Table 2:** Participant characteristics for low and high SRS-2 traits groups (*n* = 120).

	SRS-2 Low (*n* = 69)				SRS-2 High (*n* = 51)			
	%	Mean	*SD*	Range	%	Mean	*SD*	Range
Gender								
Female	59.40				58.80			
Male	40.60				31.40			
Non-binary	0				9.80			
Age		25.78	8.14	18–56		21.92	5.82	18–58
Visual Acuity at 20 ft (20/*x*)		20/18.30	4.10	20/13–20/25		20/19.41	3.88	20/13–20/25
SRS-2 total T score		51.81	4.52	40–59		69.20	7.69	60–90
Awr T		49.14	7.83	32–69		65.75	8.84	41–86
Cog T		52.97	4.97	42–63		65.57	8.49	49–90
Com T		51.45	5.60	41–70		68.80	8.61	50–90
Mot T		52.93	6.18	41–67		68.61	8.92	54–90
RRB T		51.26	5.94	40–70		69.22	10.82	53–90
SCI T		52.00	4.43	40–60		68.57	7.43	58–90

Abbreviations: SRS-2, Social Responsiveness Scale, 2nd Edition; Awr T, Social Awareness t-score; Cog T, Social Cognition t-score; Com T, Social Communication t-score; Mot T, Social Motivation t-score; RRB T, Restricted Interests and Repetitive Behaviors.

SRS-2 total raw scores and scores for individual subscales (Awr, Cog, Com, Mot, and RRB), and the Social Communication and Interaction subscale (SCI; which combines raw scores from the Awr, Cog, Com, and Mot scales) were calculated and transformed into standardized T-scores. Participants with T-scores above 59 were classified as high on autistic traits, while those below were considered low in accordance with standard practices^38^.

In the final sample (*n* = 120), fifty-one participants (42.5%) were categorized as high on autistic traits, with the remaining 69 (58%) in the low group. All seven self-identified autistic participants were in the high traits group. Additionally, 32 participants (26.7%) reported having a first- or second-degree family member with ASD, with 17 (53.1%) of them were classified as high on autistic traits. These rates align with previous research indicating prevalence of traits among 20–50% of family members of autistic individuals^39,40^.

Analyses focused on total scores categorically (low, high groups) and on a continuum. No significant differences were found between low and high trait groups in visual acuity (SRS-2: *p* > 0.14; see Table 2).

### Behavioral data processing

Behavioral results files were produced individually for each participant as .csv files using the SR Research Experiment Builder (Version 2.3.1)^41^ executable program. Preprocessing, including cleaning and averaging of the data, was done through custom R scripts run in RStudio (Version 7.1)^42^.

Participants were required to respond within the 4000 ms timeframe while the stimulus was on the screen for at least 50% of trials. Two participants were excluded for failing to meet this criterion, as they did not respond within the required timeframe for any trials. For participants with sufficient response data, to ensure responses were reflective of participants’ perception of the stimulus properties rather than random responses, a reliability measure was computed based on congruent trials (trials where the same response was expected for the global and local level). This reliability measure was equivalent to an accuracy rate for congruent trials, defined as the proportion of correct responses during congruent trials. If reports were non-random, participants should have responded more often with the correct orientation on congruent trials than might be expected by chance, calculated at 25% given that there were four response options. A reliability cutoff of 30% was then used to determine responding above chance in the current analyses, leading to the exclusion of 16 additional participants in subsequent analyses. With these 18 participants excluded, this resulted in a final sample of 120 participants. Of the included participants, the correct orientation was reported in congruent trials at a mean rate of 71.7% (*SD* = 20.9%).

For included participants, individual trials were examined, and trials with responses occurring after the stimulus duration (> 4000 ms) or faster than 300 ms were discarded (in keeping with Campana et al.^2^). Participants had an average of 23 (*SD* = 1.89) incongruent trials and average of 23 (*SD* = 1.51) congruent trials remaining. Mean reaction times were then computed for congruent and incongruent trials, and separately for global and local reports during incongruent trials.

### Pupillary data processing

#### Preprocessing

Eye tracking data were exported using Data Viewer software Version 4.3.210 (SR Research Ltd., 2020) and cleaned individually for each participant using adapted scripts from the R package “PupilPre” Version 0.6^43.^ Offscreen data were removed, blinks and artifacts were identified and corrected. After cleaning, trials missing over 50% of pupil data in baseline (500 ms before stimulus onset) or the critical window from stimulus onset to offset (0–4000 ms) were excluded. Participants had an average of 45 trials (*SD* = 4.41). Pupil size data were scaled from pixels to millimeters and baseline corrected by subtracting the averaged pupil size during the 500 ms prior to stimulus onset, according to best practices^44^. Missing values were linearly interpolated, and data were downsampled to 5 Hz, resulting in twenty 200 ms bins. Custom R scripts organized cleaned pupil data for subsequent analyses. Of the 120 participants included in the behavioral analysis, five were excluded from pupil analysis due to missing eye-tracking data for over 50% of trials in the experimental condition.

All reported pupillary analyses were performed on averaged baseline-corrected data. In past work, baseline pupil size has also been investigated in similar hierarchical processing tasks (e.g.,^33,34^); however, significant differences in baseline pupil diameter were found due to the lighting differences between the two experimental sites (*t*(113) = − 13.07, *p* < 0.001), and baseline pupil size was therefore not examined as its own metric. Preliminary analyses were conducted to examine whether testing site had an effect on general pupillary analyses; because no significant effects of site were found, this variable was not included in subsequent analyses.

#### Pupil phases (peak-to-trough measurements)

The pupillary waveform, akin to an event-related potential waveform, exhibits specific phase-based changes derived from distinct peaks and troughs. A previous study by DiCriscio et al.^34^ with adults identified four phases of the pupillary response to visual stimuli; Phase 1 (0–250 ms): pupil dilation post-stimulus onset, Phase 2 (250–1000 ms): rapid constriction, Phase 3 (1000–2000 ms): slow redilation reaching a maximum, and Phase 4 (2000–5000 ms) a decrease to the trial end. Phase-based patterns were also observable in the current data; however, visual inspection of individual waveforms revealed that key points of the response (i.e., the minimum point of constriction and the later maximum point of dilation) showed individual variability in their respective latencies. Because of this, the start and end points of the phases in the current study were flexibly defined to accommodate these individual differences (rather than sectioned by pre-defined time bins^34^).

**Fig. 5:**
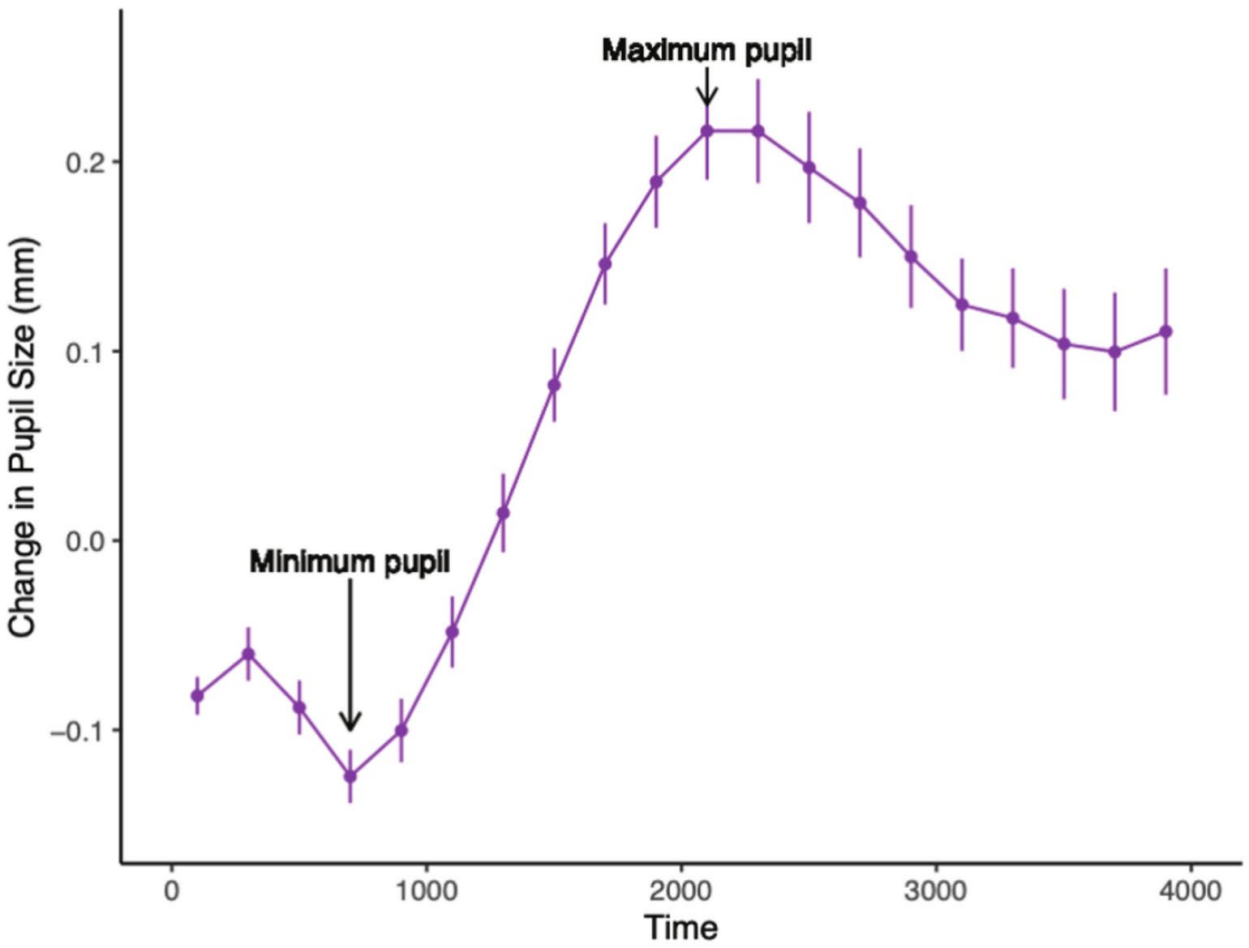
Point of minimum pupil constriction and point of maximum dilation plotted on a down-sampled (5 Hz) task-evoked pupil response from a single participant.

**Fig. 6:**
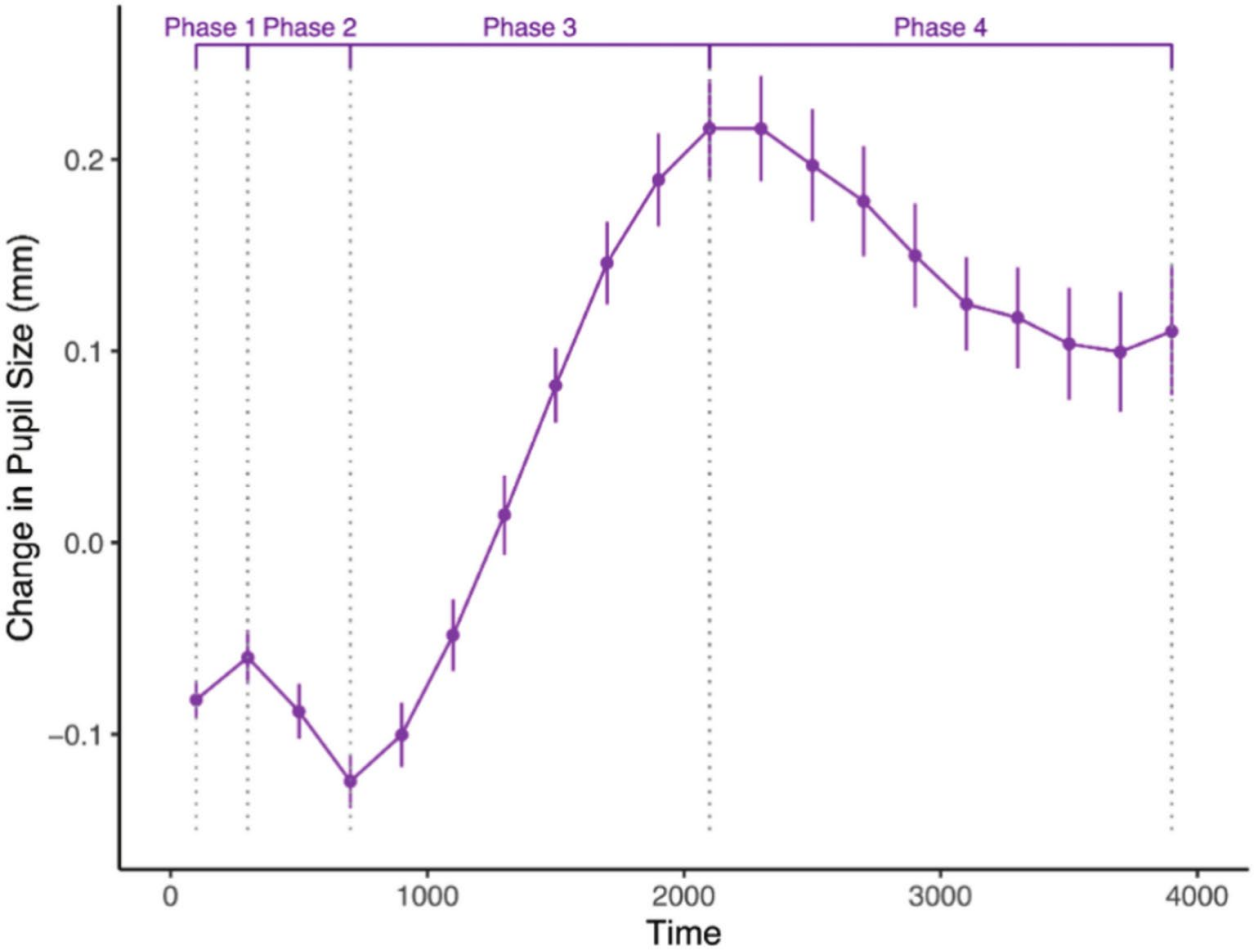
Down-sampled (5 Hz) task-evoked pupil response for one participant, divided into the four phases.

On initial examination of the pupil waveforms, most responses were characterized by initial constriction between 800 and 1200 ms, followed by dilation around 2000–2400 ms (e.g., see Fig. 5). As a first pass, an automated selection process identified Phases 1–4 based on the criteria of DiCriscio et al.^34^. All waveforms were then visually inspected, and the search window was made wider or shifted if necessary to facilitate the identification of the “true” peak and dip. Phase magnitude was then calculated by taking the difference in pupil diameter between the start and end of the phase (see Fig. 6). This approach resembles peak-to-trough measurement in stimulus/task-evoked electroencephalography waveforms. However, rather than taking the absolute value of the change, as is commonly done with neural waveforms, directionality (+/−) of the change was maintained, following prior methodology (i.e.,^34^). With start of phase subtracted from end of phase, positive values generally indicate dilation during a given phase, and negative values indicate constriction. In the current sample, as well as in past work^34^, a characteristic pupil waveform generally included an amplitude change that was positive for Phase 1, negative for Phase 2, positive for Phase 3, and negative for Phase 4.

#### Trajectory groups

We used finite mixture modeling to establish pupil response trajectories using a censored normal (CNORM) distribution, employing the group-based trajectory modeling framework^45^. This approach combined single group models into a common multiple-group model structure to identify distinct subpopulations within the data. Participants’ averaged downsampled pupillary waveforms were first visually inspected at the individual level, revealing patterns of early constriction and later dilation. This led to the hypothesis of two distinct latent classes, informing our model under the assumption that the data were generated by two underlying distributions. Trajectory analyses were performed in SAS Version 9.4^46^ using the TRAJ procedure^47^ and an adaptation of a script used in DiCriscio et al.^30^ TRAJ uses a discrete mixture model to cluster time series data into subgroups presumed to exist within the aggregate distribution.

This analysis modeled time bin trends as polynomial functions, specifically testing intercept, linear, and quadratic terms. Initial model selection was informed by our hypothesis of two trajectory groups based on visual inspection of each participant’s downsampled, averaged waveform, and the literature^30,33^. Given the larger sample in our study, we assessed the possibility of additional trajectory groups. Guided by Bayesian Information Criteria (BIC), we compared models with three and four trajectories, with lower BIC indicating better fit (see Table 3). The final model was selected that balanced the goodness-of-fit with model complexity.

**Table 3:** Model comparison using Bayesian Information Criterion (BIC).

Model	Number of groups	BIC
1	2	2365.47
2	3	2704.28
3	4	2860.58

Smaller BIC indicates a better fit.

### Statistical analysis

Statistical analyses were conducted using IBM SPSS software^48^ (Version 29). To examine effects of autistic traits, two approaches were used: (1) traits group (low, high) was included as a fixed factor in linear mixed models, and (2) bivariate correlations were conducted with SRS-2 total T scores. In cases of significant correlational findings with SRS-2 total T scores, follow-up correlations were conducted with the individual SRS-2 subscale T scores.

Linear mixed modeling (LMM) was used to examine within-participant variability relative to between-participants variability, with participants included as a random effect of intercept and maximum restricted likelihood estimation utilized. Fixed factors were pre-determined based on our theoretically driven hypotheses and to enable direct comparison with Campana et al.^2^ Regression coefficients and model summary statistics of hierarchically built models, starting from the null model, are presented in Supplemental Materials in corresponding tables to assess the influence of each factor on model fit. Chi-squared likelihood ratio tests (change in − 2LL) were used to assess the improvement of the fit with the addition of new fixed factors in the hierarchical model. As factors were added, we expected smaller − 2LL, which indicates an improved fit. As all terms were of theoretical interest, the in-text results focus on the saturated model, with main effects and interactions reported. Even in cases where individual fixed factors did not significantly improve model fit, their inclusion supports the broader theoretical framework and interpretation of the findings.

Additional statistical tests included independent samples *t-*tests. Effect sizes for pairwise comparisons were calculated using Hedge’s* g*, with values of 0.2, 0.5, and 0.8 or greater representing small, medium, and large effects, respectively.

## Results

### Behavioral measures

Preliminary LMM analysis revealed an effect of congruency on reaction time results, showing that participants were significantly faster to report orientation in congruent (*M* = 1846 ms, *SD* = 460) compared to incongruent trials (*M* = 1923 ms, *SD* = 435), *F*(120) = 15.21, *p* < 0.001. Subsequent analyses examined default reports and reaction time (RT) for incongruent trials only, where global and local level responses are in conflict.

#### Default reports

LMM was conducted with report type (global, local) and traits group (low, high) as fixed factors to examine report rates of the global and local orientation in response to incongruent trials. Results showed that participants responded with the global orientation (*M* = 44.3%, *SD* = 32.7%) significantly more often than with the local orientation (*M* = 30.5%, *SD* = 24.5%), *F*(246) = 14.95, *p* < 0.001. There was no main effect of group (*p* = 0.66) nor interaction between report type and group (*p* = 0.58). Bivariate correlations with total scores from the SRS-2 showed no relationship between autistic traits and global or local report rates (*p*s > 0.26). Model fit significantly improved with the addition of report type (Δ-2LL = 13.39, Δ*df* = 1, *p* < 0.001) but not with the inclusion of traits group as a fixed factor (Δ-2LL = 1.2, Δ*df* = 2, *p* = 0.55). Supplementary Table 1 presents regression coefficients for hierarchical LMM of report rates.

#### Reaction time

LMM with report type (global, local) and traits group (low, high) as fixed factors showed a significant main effect on RT, with faster responding when reporting the global orientation than when reporting the local orientation (global: *M* = 1910 ms, *SD* = 524; local: *M* = 2030 ms, *SD* = 513; *F*(1, 111.28) = 4.09, *p* = 0.046). There was no significant effect of traits group (*p* = 0.125) nor interaction between report type and traits group (*p* = 0.458). Model fit was not significantly improved by the addition of report type (Δ-2LL = 3.49, Δ*df* = 2, *p* = 0.06) or traits group as fixed factors (Δ-2LL = 2.76, Δ*df* = 2, *p* = 0.25). Supplementary Table 2 shows regression coefficients for hierarchical LMM of reaction time. Bivariate correlations showed no association between SRS-2 total score and RT for global or local reports (*p*s > 0.07).

### Pupillary measures

Preliminary analyses revealed no effect of congruency on the pupillary measures results. Subsequent results are therefore reported based on averaged responses to all valid trials, including both congruent and incongruent.

#### Pupil phase amplitudes (peak-to-trough measurements)

**Fig. 7:**
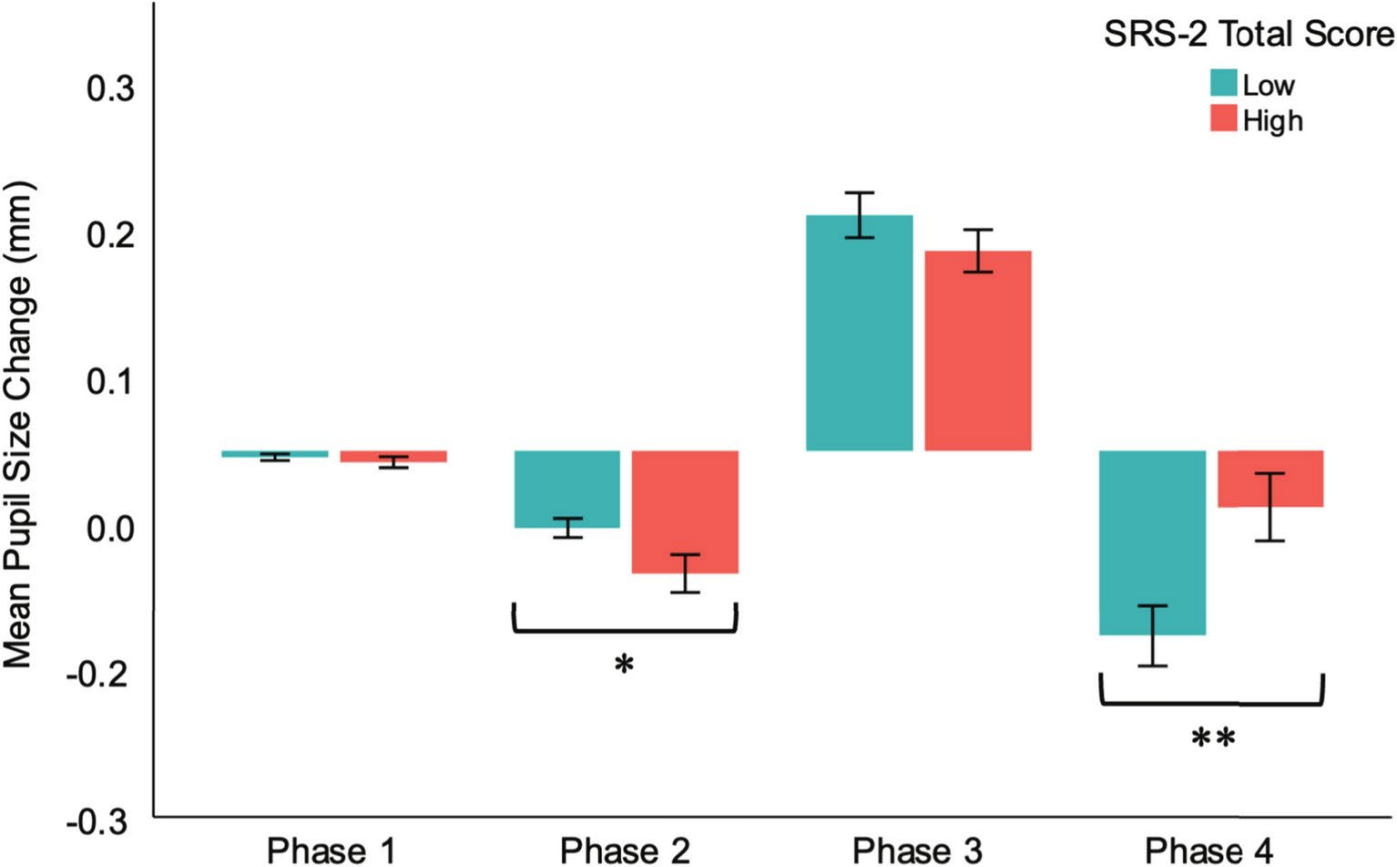
Differences in pupil size change between low and high SRS-2 traits groups in Phases 2 and 4, * *p* < .05, ** *p* < .01.

LMM with Phase (1, 2, 3, 4) and traits group (low, high) as fixed factors showed a main effect of phase, *F*(3, 452) = 110.06, *p* < 0.001, with significant differences in pupil size changes from all phases except between Phase 3 and 4. There was a significant phase by traits group interaction, *F*(3, 452) = 7.47, *p* < 0.001 (see Fig. 7), and follow up pairwise comparisons showed that the high traits group had significantly greater constriction in Phase 2 (*M* = -0.08 mm, *SD* = 0.09) than the low traits group (*M* = -0.05 mm, *SD* = 0.02; *t*(72.22) = 2.15, *p* = 0.035, Hedge’s *g* = 0.42), but showed significantly less constriction in the return to baseline from maximum pupil during Phase 4 (Low: *M* = -0.13 mm, *SD* = 0.17; High: *M* = -0.04 mm, *SD* = 0.16), *t*(113) = -2.80, *p* = 0.006, Hedge’s *g* = 0.53). Model fit significantly improved with the addition of Phase (Δ-2LL = 251.28, Δ*df* = 4, *p* < 0.001) and traits group (Δ-2LL = 22.38, Δ*df* = 4, *p* < 0.001) as fixed factors. Supplementary Table 3 shows regression coefficients for hierarchical LMM of pupil phase amplitudes.

Correlational analyses showed that SRS-2 total scores were negatively associated with Phase 2 change, *r*(113) = -0.298, *p* = 0.001, and positively associated with Phase 4 change, *r*(113) = 0.276, *p* = 0.003, suggesting that individuals with higher levels of autistic traits showed more constriction during Phase 2 and less during Phase 4. No significant correlations were found between autistic traits and Phase 1 or Phase 3 (SRS-2: *p*s > 0.12). In follow-up analyses examining correlations with subscale scores of the SRS-2, all subscales (RRB, Mot, Com, Cog, Awr) and the SCI composite scores were negatively associated with Phase 2 change (*r*s < -0.21, *p*s < 0.04) and positively associated with Phase 4 change (*r*s > 0.21, *p* < 0.03).

#### Trajectory groups

**Fig. 8:**
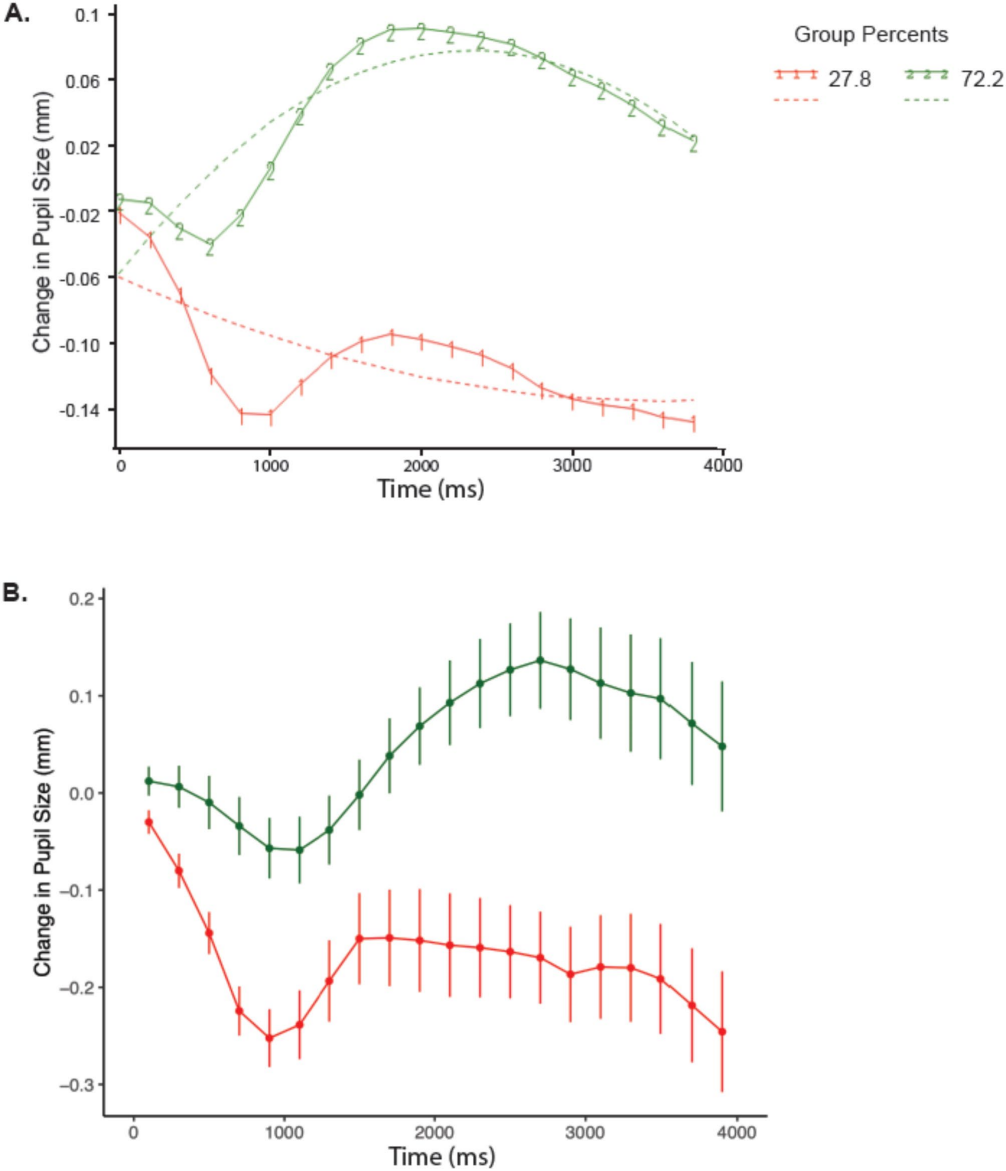
(**A**) Task-evoked pupil response trajectories for Trajectory 1 (red) and Trajectory 2 (green) groups. Solid lines represent the mean of the trajectory group. Dashed lines represent the modeled trajectory following a quadratic function. (**B**) Task-evoked pupil waveform from a single participant in the Trajectory 1 group (red) and in the Trajectory 2 group (green). Error bars represent 1 *SE* above and below the mean.

The final model was specified as a CNORM model with polynomial trends up to the quadratic level. Two quadratic functions were determined to offer the best model fit based on BIC (see Table 3). The analysis identified two latent trajectory groups characterized by quadratic functions based on significant parameter estimates for intercept, linear, and quadratic components. These parameters are summarized in Table 4. Participants were assigned to the group to which they had the higher probability of belonging (see Fig. 8A). Trajectory 1 (in red; Fig. 8A) accounted for responses from 27.8% of participants (*n* = 32). Trajectory 2 (in green; Fig. 8A) accounted for responses from 72.2% of participants (*n* = 83). Figure 8B shows example waveforms from individual participants in each trajectory group. Pearson’s Chi-square tests of independence were conducted to determine if level of autistic traits was associated with pupil response trajectory patterns. A disproportionally higher number of participants with low traits showed a more dilated pupil response (Trajectory 2,* n* = 53), χ2(1) = 5.10, *p* = 0.024 (see Table 5).

**Table 4:** Model indices for trajectory analysis.

Group	Parameter	Estimate	Standard Error	*t* for *H*_0_: Parameter = 0^a^
1	Intercept	− 0.059	0.009	− 6.516***
	Linear	− 0.008	0.002	− 3.801***
	Quadratic	0.001	0.001	2.083*
2	Intercept	− 0.057	0.006	− 10.213***
	Linear	0.023	0.001	16.577***
	Quadratic	− 0.001	0.001	− 14.035***
Sigma		0.083	0.001	67.613***

^a^Results of a *t*-test assessing whether each parameter significantly differs from zero under the null hypothesis (*H*_0_). Parameters with *p* < .05 meaningfully contribute to the model.

* *p* < .05. ** *p* < .01. *** *p* < .001.

**Table 5:** Trajectory group membership for participants low and high on autistic traits on the SRS-2.

	SRS-2 low	SRS-2 high	Total
Trajectory 1	13	19	32
Trajectory 2	53	30	83
Total	66	49	115

## Discussion

The current study examined behavioral and pupillary responses during a hierarchical visual processing task in a sample of adults with varying levels of autistic traits. This study is novel in its use of well-controlled hierarchical stimuli not yet used to examine visual processing in relation to autistic traits, employing both behavioral performance metrics and advanced analytic approaches to the transient pupillary waveform to enhance the detection of subtle processing differences.

We found that overall, participants spontaneously reported the global orientation significantly more often than they reported the local orientation, replicating findings from Campana et al.^2^ and providing additional support for global default processing. Furthermore, when participants spontaneously reported the global orientation, their reaction time was faster than when they reported the local orientation, suggesting the global orientation was consciously perceived faster than the local orientation, even though initial processing of the local information was necessary for the global orientation to be perceived.

The hypothesis that high levels of autistic traits would be associated with more default reports of the local orientation (as suggested by findings from Koldewyn et al.^11^) was not supported by the results. In the current study, trait level had no effect on default reports, suggesting that both adults with low and high levels of autistic traits exhibit global default processing.

For pupillary responses, it was hypothesized that the high traits group would show a more constricted response in the early part of the response compared to the low traits group, possibly reflecting a more locally-oriented visual strategy defined by narrower attentional focus (i.e.,^34^). The results supported this hypothesis, showing an effect of traits group on pupil responses. During the early dip in the response (Phase 2), the high traits group was more constricted than the low traits group, and the high traits group remained relatively constricted throughout the trial and showed a smaller return to baseline during Phase 4. Additionally, correlational analyses showed that more constriction during Phase 2 and less constriction during Phase 4 was associated with higher levels of autistic traits. Trajectory analyses showed that the low traits group was disproportionately represented in a dilated trajectory response group.

Taken together, this study showed that while behavioral responses were similar regardless of autistic traits, pupil responses suggest differential processing between traits groups. This indicates that although visual strategies may differ, both groups ultimately arrive at the same conclusion with default global processing. High autistic trait individuals may use a different and potentially more locally oriented visual strategy, reflecting a more narrow breadth of attentional focus, to achieve a global perceptual response despite having similar behavioral reactions to those with lower levels of autistic traits who may have a wider breadth of attentional focus.

As suggested by Turi et al.^35^ and Koldewyn et al.^11^ and supported by the current study, free-viewing tasks that reflect default processing have the potential to reveal subtle aspects of visual processing strategies preferred by autistic individuals and/or those with high autistic traits. It is important to explore this further, as these pupil response findings suggest an attentional breadth mechanism may be responsible for more narrowly gating visual processing in those with higher levels of autistic traits. This could be a potential mechanism by which global/local processing differences might emerge. Further, it may be that this processing strategy is a default, but as work by Turi et al.^35^ and Koldewyn et al.^11^ suggest, it is not mandatory, and individuals displaying a locally-oriented default can shift their processing strategy during directed tasks.

It should be noted that this study did not include analysis of baseline pupil diameter (the pupil size immediately preceding each stimulus) as its own metric of interest due to differences in ambient lighting at the two testing sites used. Although both testing sites had similar conditions, it was not possible to control the lighting to the exact lux. However, because all reported pupillary analyses were performed on baseline-corrected data, this ambient light difference should not have affected the presented results, as the task-evoked pupillary response has been found to be independent of baseline pupil size (e.g.,^49^).

Further, while past findings have shown both task (global vs. local^34^) and group effects (autistic vs. nonautistic^50^) on intertrial pupil size, future studies may benefit from focusing on resting-state pupil size as a more meaningful metric. Resting-state pupil size (defined as pupil size during free viewing of visual stimuli with no task demands present), reflects tonic arousal and attentional states and has been linked to task performance. For instance, larger resting state pupil size in autistic children has been associated with superior performance in visual tasks^51^.

Additionally, while we applied baseline correction to reduce trial-to-trial fluctuations in pupil size, we chose not to include baseline in the models, following guidance from Mathôt and Vilotijević^44^. They caution that modeling baseline values can introduce artifacts, such as negative correlations with task-evoked responses due to regression to the mean, and amplify noise, potentially obscuring meaningful effects. By focusing solely on baseline-corrected pupil responses, we aimed to avoid these pitfalls while adhering to best practices for robust and interpretable analyses^44^. This approach ensures that our results reflect genuine task-evoked changes, free from confounding influences of baseline variability. Relatedly, while baseline correction should normalize responses, the constriction observed in Phase 4 may have influenced the baseline of the subsequent trial. Future iterations should extend the intertrial interval to minimize this potential effect.

It is important to underscore that the group effects examined in the current study focused on the impact of autistic traits on hierarchical perception, rather than exploring the effects of ASD. While this study did include autistic individuals, the group was small (*n* = 7) and formal ASD diagnostic tests were not administered — participants were instead asked to self-identify. Consequently, we cannot confirm that these individuals had formal diagnoses. Additionally, it is possible that some participants who identified as nonautistic were either undiagnosed or chose not to disclose their diagnosis. It is therefore not possible to generalize findings about autistic traits to conclusions about autistic individuals (for further discussion, see^52^). Future work should seek to study this paradigm in a larger sample of individuals with clinician-confirmed autism diagnoses to better understand how visual processing strategies might relate to core features of autism.

Further, it is worth considering findings from Nyström et al.^53,54^, who demonstrated that infants with an elevated likelihood for autism exhibited more pronounced pupillary light reflex compared to low likelihood infants, suggesting atypical autonomic regulation or sensory responsiveness. Although the pupillary light reflex represents a distinct physiological response compared to task-evoked pupillary changes, these findings suggest that individuals with high autistic traits or at elevated likelihood for autism show differences at multiple stages of sensory and attentional processing.

Overall, the findings from the current study can be taken as further support for global precedence and global default processing; compared to local-level information, global-level information was processed more quickly and automatically by participants, regardless of level of autistic traits. When examining pupillary responses, a constricted pattern was associated with higher autistic traits, and individuals low on autistic traits were more likely to show a dilated trajectory, suggesting that differential processing strategies related to attentional breadth may be associated with differences in visual processing. Notably, the lack of behavioral differences here, despite distinct pupillary responses, suggests that individuals with varying levels of autistic traits may rely on different visual processing strategies to achieve similar outcomes. These distinct strategies could influence broader behavioral patterns when applied to real world stimuli and could also indicate underlying neural processing differences.

Specifically, we are curious to know how these processing patterns might relate to ASD phenotypes and characteristics, such as attention to detail, insistence on sameness, and rigidity, and how they may reflect distinct processing styles influencing everyday experiences of individuals with elevated autistic traits. A narrow attentional focus could facilitate superior attention to detail, a strength in certain contexts, such as tasks requiring high precision. However, this same strategy might pose challenges in situations that require rapid synthesis of global information, such as navigating complex social interactions or interpreting contextual cues in dynamic environments. We propose that a narrower attentional breadth might gate perception in a way that amplifies the significance of small environmental changes, potentially contributing to behaviors such as insistence on sameness and rigidity. While highly speculative, this interpretation warrants further exploration in future research. In addition to including a large sample of autistic individuals with a confirmed diagnosis, future work should investigate whether the identified pupil trajectory groups correspond to varying levels of adaptive functioning, sensory sensitivities, or specific behavioral phenotypes.

Identifying individuals who exhibit a constricted pupil trajectory could offer valuable insights into subtypes of autism characterized by this processing style. This, in turn, may inform the development of targeted interventions aimed at expanding attentional focus. Such interventions could include cognitive training tasks designed to enhance global processing and promote flexible attention, or structured activities that encourage individuals to integrate broader contextual information. Tailoring these approaches to the unique needs of this subgroup could support improved adaptive functioning and greater flexibility in responding to environmental changes. Equally important, understanding the behavior of individuals who exhibit a narrower focus may foster greater acceptance and appreciation of this distinct way of processing the world. Such understanding could encourage the development of thoughtful environmental adaptations and a more empathetic acknowledgment of rigid and sameness behaviors, which might otherwise be misunderstood.

Although the current study was unable to provide a definitive mechanistic explanation for visual processing in the context of autistic traits, future studies can build on this approach to continue exploring the role attention might play in neurodivergent visual processing.

## Supplementary Information

Supplementary Information.

## Data Availability

Data is available upon request by contacting the first author (chloebrittenham@gmail.com).
