# Supplementary material for: Task-evoked pupil responses during free-viewing of hierarchical figures in relation to autistic traits in adults
Source: Sci Rep. 2025 Mar 21;15:9778. doi: 10.1038/s41598-025-92904-x (PMC11928501; doi:10.1038/s41598-025-92904-x)
Supplement: Supplementary file 1 — Supplementary Information. [file 41598_2025_92904_MOESM1_ESM.docx]

**Supplemental Materials**

Supplementary Table 1. Linear mixed-effects models for report rate.

|  | Regression coefficients (estimate of fixed effects) | | |
| --- | --- | --- | --- |
| Effect | Model 1 (null) | Model 2 | Model 3 |
| Intercept | .37(0.19)*** | .31(.03)*** | .28(.04)*** |
| Main effects |  |  |  |
| Report type |  |  |  |
| Global | — | .138(.04)*** | .19(.06)** |
| Local | — | 0^a^ | 0^a^ |
| Traits group |  |  |  |
| Low | — | — | .05(.05) |
| High | — | — | 0^a^ |
| Interactions |  |  |  |
| Global x Low | — | — | -.08(.08) |
| Global x High | — | — | 0^a^ |
| Local x Low | — | — | 0^a^ |
| Local x High | — | — | 0^a^ |
| Model Summary -2LL^b^ | 97.73 | 84.34*** | 83.14 |
| Estimated Parameters | 3 | 4 | 6 |

Note. Standard errors for parameter estimates are listed in parentheses.

^a^ The parameter is set to zero because it is redundant. Cross-level interactions with redundant parameters are excluded from the table.

^b^ -2LL; -2 log-likelihood is a measure of how well the model fits the data. Smaller numbers reflect a better fit.

* *p* < .05. ** *p* < .01. *** *p* < .001.

Supplementary Table 2. Linear mixed-effects models for reaction time.

|  | Regression coefficients (estimate of fixed effects) | | |
| --- | --- | --- | --- |
| Effect | Model 1 (null) | Model 2 | Model 3 |
| Intercept | 1957.30(41.87)*** | 2008.13 (49.6) *** | 2107.60(77.28)*** |
| Main effects |  |  |  |
| Report type |  |  |  |
| Global | — | -93.82(50.0) | -141.35(78.80) |
| Local | — | 0^a^ | 0^a^ |
| Traits group |  |  |  |
| Low | — | — | -166.67(100.21) |
| High | — | — | 0^a^ |
| Interactions |  |  |  |
| Global x Low | — | — | 76.11(102.13) |
| Global x High | — | — | 0^a^ |
| Local x Low | — | — | 0^a^ |
| Local x High | — | — | 0^a^ |
| Model Summary -2LL^b^ | 3393.28 | 3389.79 | 3387.03 |
| Estimated Parameters | 3 | 4 | 6 |

Note. Standard errors for parameter estimates are listed in parentheses.

^a^ The parameter is set to zero because it is redundant. Cross-level interactions with redundant parameters are excluded from the table.

^b^ -2LL; -2 log-likelihood is a measure of how well the model fits the data. Smaller numbers reflect a better fit.

* *p* < .05. ** *p* < .01. *** *p* < .001.

Supplementary Table 3. Linear mixed-effects models for pupil phase amplitude.

|  | Regression coefficients (estimate of fixed effects) | | |
| --- | --- | --- | --- |
| Effect | Model 1 (null) | Model 2 | Model 3 |
| Intercept | -.003(.007) | -.09(.01)*** | -.04(.02)* |
| Main effects |  |  |  |
| Phase |  |  |  |
| Phase 1 | — | -.00(.01)*** | .03(.02) |
| Phase 2 | — | .08(.01) | -.05(.02)* |
| Phase 3 | — | .02(.01)*** | .18(.02)*** |
| Phase 4 | — | 0^a^ | 0^a^ |
| Traits group |  |  |  |
| Low | — | — | .09(.02)*** |
| High | — | — | 0^a^ |
| Interactions |  |  |  |
| Phase 1 x Low | — | — | .09(.03)** |
| Phase 1 x High | — | — | 0^a^ |
| Phase 2 x Low | — | — | .12(.03)*** |
| Phase 2 x High | — | — | 0^a^ |
| Phase 3 x Low |  |  | .11(.03)*** |
| Phase 3 x High |  |  | 0^a^ |
| Phase 4 x Low |  |  | 0^a^ |
| Phase 4 x High |  |  | 0^a^ |
| Model Summary -2LL^b^ | -479.07 | -730.36*** | -752.73*** |
| Estimated Parameters | 2 | 6 | 10 |

Note. Standard errors for parameter estimates are listed in parentheses.

^a^ The parameter is set to zero because it is redundant. Cross-level interactions with redundant parameters are excluded from the table.

^b^ -2LL; -2 log-likelihood is a measure of how well the model fits the data. Smaller numbers reflect a better fit.

* *p* < .05. ** *p* < .01. *** *p* < .001.
